# Harmonics of Circadian Gene Transcription in Mammals

**DOI:** 10.1371/journal.pgen.1000442

**Published:** 2009-04-03

**Authors:** Michael E. Hughes, Luciano DiTacchio, Kevin R. Hayes, Christopher Vollmers, S. Pulivarthy, Julie E. Baggs, Satchidananda Panda, John B. Hogenesch

**Affiliations:** 1 Department of Pharmacology, Institute for Translational Medicine and Therapeutics, University of Pennsylvania School of Medicine, Philadelphia, Pennsylvania, United States of America; 2 Regulatory Biology, Salk Institute for Biological Studies, La Jolla, California, United States of America; Stanford University School of Medicine, United States of America

## Abstract

The circadian clock is a molecular and cellular oscillator found in most mammalian tissues that regulates rhythmic physiology and behavior. Numerous investigations have addressed the contribution of circadian rhythmicity to cellular, organ, and organismal physiology. We recently developed a method to look at transcriptional oscillations with unprecedented precision and accuracy using high-density time sampling. Here, we report a comparison of oscillating transcription from mouse liver, NIH3T3, and U2OS cells. Several surprising observations resulted from this study, including a 100-fold difference in the number of cycling transcripts in autonomous cellular models of the oscillator versus tissues harvested from intact mice. Strikingly, we found two clusters of genes that cycle at the second and third harmonic of circadian rhythmicity in liver, but not cultured cells. Validation experiments show that 12-hour oscillatory transcripts occur in several other peripheral tissues as well including heart, kidney, and lungs. These harmonics are lost ex vivo, as well as under restricted feeding conditions. Taken in sum, these studies illustrate the importance of time sampling with respect to multiple testing, suggest caution in use of autonomous cellular models to study clock output, and demonstrate the existence of harmonics of circadian gene expression in the mouse.

## Author Summary

Circadian rhythms confer adaptive advantages by allowing organisms to anticipate daily changes in their environment. Over the last few years, many groups have used microarray technology to systematically identify genes under circadian regulation. We have extended on these studies by profiling the circadian transcriptome from the mouse liver and two immortalized cell lines at an unprecedentedly high temporal resolution. We identified over 3,000 different transcripts in the mouse liver that cycle with a period length of approximately 24 hours. To our surprise, we also identified two classes of genes which cycle with period lengths of 12 and 8 hours; i.e., harmonics of the circadian clock. Importantly, we were able to identify harmonics in five other tissue types; however, these rhythms were undetectable in disassociated cells. Moreover, harmonics were lost in the liver when mice are subjected to restricted feeding, suggesting that at least one component of circadian harmonics is driven by feeding.

## Introduction

Circadian rhythms are daily, 24-hour (h) oscillations in physiology and behavior such as food consumption, blood pressure, metabolism, body temperature, and locomotor activity [1],[2]. These rhythms are thought to give an adaptive advantage by allowing an organism to anticipate changes in the environment and regulate physiology accordingly. Moreover, disruptions of circadian rhythms contribute to numerous pathologies including metabolic and cardiovascular disorders, cancer, and aging [3]–[5]. A molecular and cellular clock composed of transcriptional feedback loops generates these oscillations [6]. The central loci of the mammalian clock are two small clusters of hypothalamic neurons called the suprachiasmatic nuclei (SCN), which constitute the master pacemaker that orchestrates rhythmic patterns of behavior and physiology throughout the organism [7]. Remarkably, most tissues in the body also contain autonomous circadian clocks that are necessary for the rhythmic expression of clock output genes [8] and capable of sustained oscillations outside of the body (e.g. [9]). These peripheral clocks are principally regulated by stimuli downstream from the SCN, and are entrained by the SCN via a number of different physiological signals such as glucocorticoid production, core body temperature, or cAMP input (e.g. [7],[10]).

Rhythmic physiology is thought to manifest from the transcriptional output of core oscillator components. Consequently, studies have been performed in several model systems to identify rhythmically expressed genes in both central and peripheral tissues [8], [11]–[21]. One consistent observation is that the vast majority of circadian transcriptional output is tissue-, and not locus-, specific, implying that both local and systemic cues heavily influence circadian output. In order to more fully understand the mechanism by which local and systemic signals translate into rhythms of physiology and behavior, a detailed understanding of the circadian transcriptome is necessary. To address this question, we have developed a high resolution temporal profiling experimental design in which samples are taken every hour for 48 hours and subjected to rigorous statistical analysis. This approach has the capacity to identify rhythmic output genes with precision and accuracy. We applied this method to the study of gene transcription in the liver , an organ system that receives and integrates systemic cues, as well as synchronized NIH3T3 and U2OS cells, conventional models of the autonomous cellular oscillator [22],[23].

Here we report the identification of thousands of circadian transcripts in the mouse liver. Surprisingly, using identical statistical methods dramatically fewer cycling transcripts were identified from two models of the autonomous circadian clock, NIH3T3 and U2OS cells. In addition, we found hundreds of transcripts in the liver that cycle at the second and third harmonic of circadian oscillations. Like circadian genes, these ultradian rhythms are severely dampened in *ex vivo* hepatocytes. Moreover, these rhythms are shifted in a restricted feeding paradigm, demonstrating their responsiveness to systemic cues.

## Results

Wildtype C57BL/6J mice were entrained to a 12 h light, 12 h dark (LD 12∶12) environment before being released into constant darkness. Starting 18 h after the first subjective day (CT18), liver samples from 3–5 mice per time point were collected every hour for 48 h. In parallel, we collected a 48 h time course from two different cellular models of the circadian clock in order to study circadian output in the absence of systemic, circadian cues. After synchronization by forskolin shock, NIH3T3 cells were sampled every hour for 48 h, starting 20 h after synchronization. Likewise, a human osteosarcoma cell line, U2OS, was synchronized with dexamethasone and samples were collected every hour for 48 h, starting 24 h after shock. To confirm that these cells were properly synchronized, parallel cell cultures were transfected either transiently (NIH3T3) or stably (U2OS) with a circadian reporter gene, *Bmal1:luciferase* (*Bmal1:Luc*) and imaged every 10 minutes for several days to validate synchronization and rhythmicity (Figure S1). Total RNA was purified from these samples and Affymetrix arrays were used to assess global gene expression.

To account for mode failure, two different statistical algorithms were then used to identify rhythmically expressed transcripts as previously described [24]. The first algorithm, COSOPT [25], measures the goodness-of-fit between experimental data and a series of cosine curves with varying phases and period lengths. p-values are then calculated by scrambling the experimental data and re-fitting it to cosine curves in order to determine the probability that the observed data matches a cosine curve by chance alone. The second algorithm, Fisher's G-test [26], uses Fourier transforms to systematically screen experimental data for sinusoidal components. The probability (and thus, the significance) of any observed periodicity can then be tested using Fisher's g-statistic. Importantly, neither algorithm is sensitive to amplitude nor are they intrinsically biased towards any single period length, and they work with different underlying principles minimizing the risk of mode failure.

**Figure 1:**
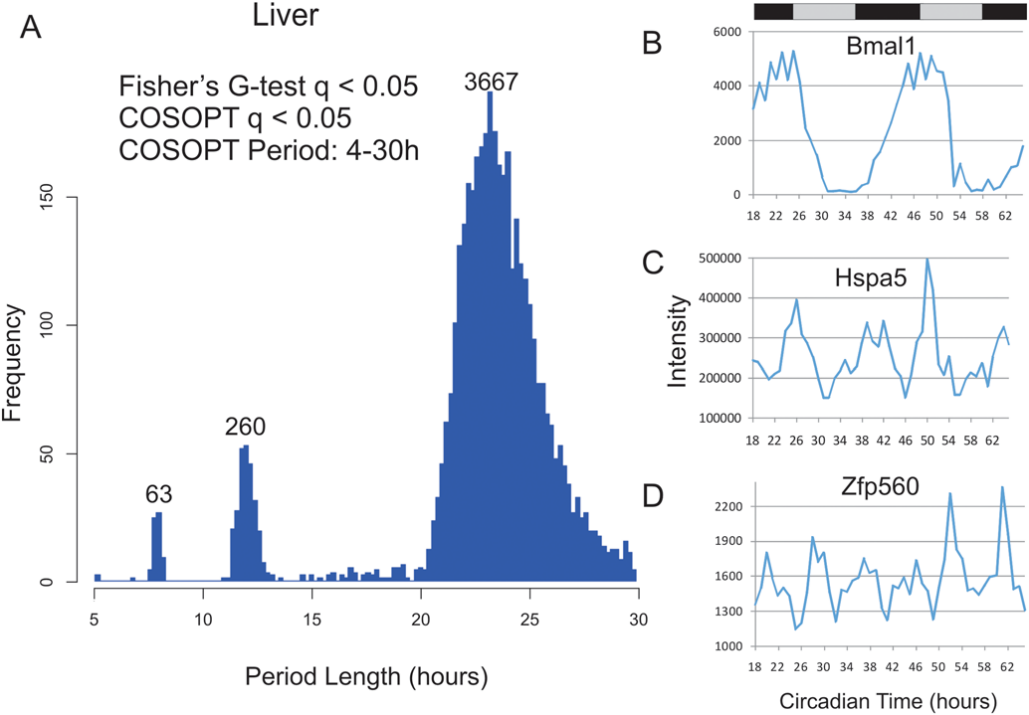
High resolution profiling in the liver identified circadian and sub-ciradian rhythms. Liver samples were collected every hour for 48 h and analyzed with Affymetrix expression arrays. Rhythmic genes were identified using both COSOPT and Fisher's G-test at a false-discovery rate of <0.05. The period length of every rhythmic transcript was plotted as a histogram; clusters of rhythmic genes with period lengths of approximately 24 (>20 and <30 hours), 12 (>10 and <14 hours) and 8-hours (>7 and <9) were observed (A). In panels B–D, the microarray intensity from three examples was plotted against CT time. Bmal1 (B), Hspa5 (C), and Zfp560 (D) expression profiles demonstrate 24, 12, and 8 h period lengths, respectively.

**Figure 2:**
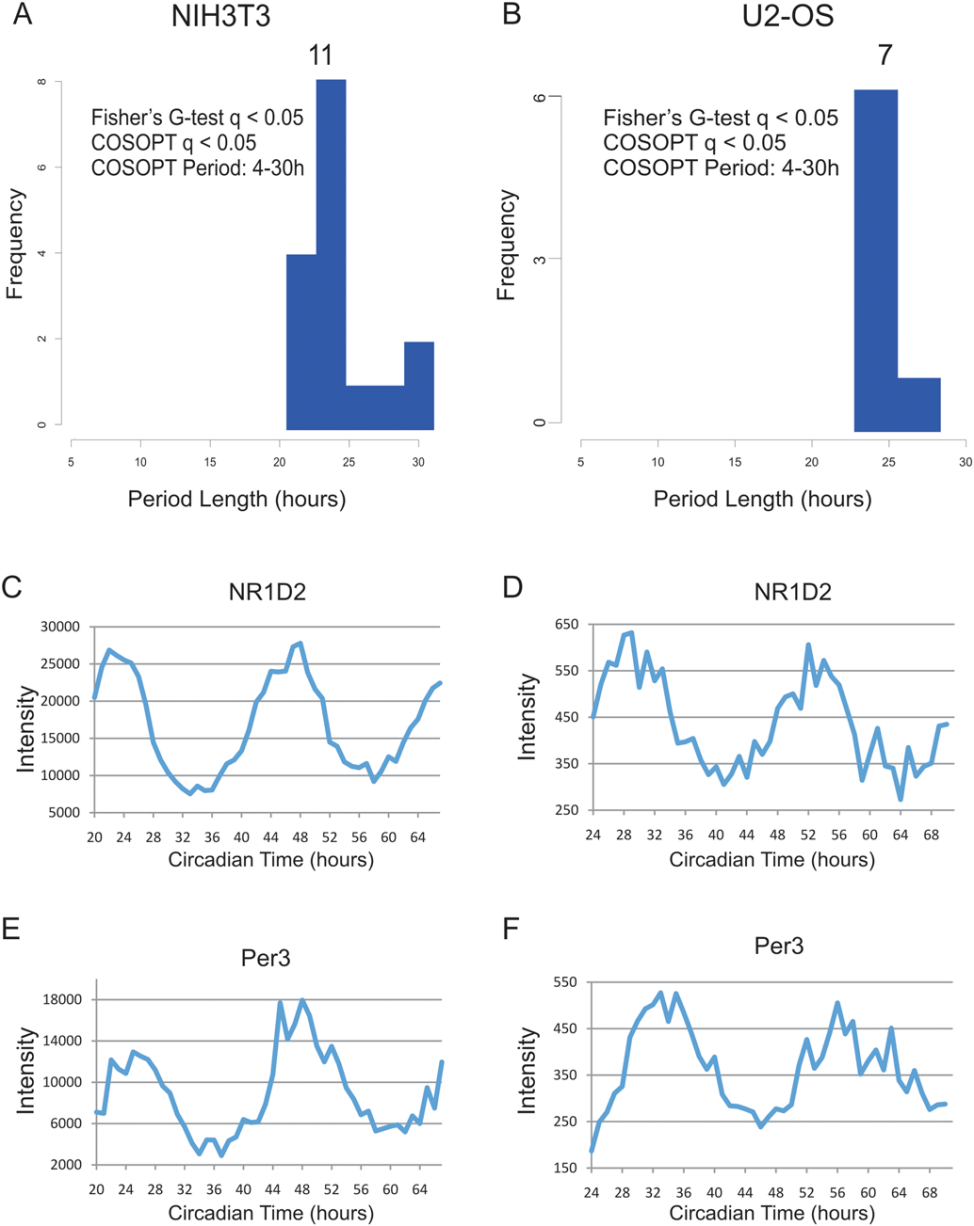
High temporal resolution profiling of NIH3T3 and U2OS cells reveals severely dampened circadian output. NIH3T3 and U2OS cells were grown to confluence and shocked with either forskolin (NIH3T3) or dexamethasone (U2OS) to synchronize their circadian clocks. mRNA samples were collected every h for 48 h and profiled on Affymetrix expression arrays. Rhythmic genes were identified using both COSOPT and Fisher's G-test at a false-discovery rate of <0.05. The period length of every rhythmic transcript was plotted as a histogram (A–B). To demonstrate that core clock genes cycle well in these data sets, panels C–F show the microarray intensity from two representative genes was plotted against CT time for both NIH3T3 and U2OS cells. NR1D2 (C–D) and Per3 (E–F) expression profiles show examples of cycling 24 h genes.

These tests were corrected for multiple comparisons post hoc using the method described by Storey and colleagues[27],[28]. Briefly, by examining the distribution of p-values from a given data set, an estimate of the proportion that are truly non-rhythmic can be derived. Using this approach to model the rate of false-discoveries, the p-value for each transcript, which estimates the frequency that a truly null observation will be labeled as significant, can be converted to a more stringent q-value which instead estimates the frequency that significant observations are truly non-rhythmic. At a false discovery rate [27] of <0.05, over 3000 transcripts were found to oscillate by both statistical tests in liver, while fewer than a dozen were found in NIH3T3 and U2OS cells. As expected, the majority of cycling transcripts from liver (and all from NIH3T3 and U2OS cells) had period lengths of approximately 24 h (Figure 1A, Figure 2A and B, Table 1).

**Table 1:** Summary of COSOPT and Fisher G stats.

Period Length	q-value	Fisher's G	COSOPT	both
**>20 and <30 Hours**	**q<0.1**	5136	6405	4507
	**q<0.05**	4148	5282	3667
	**q<0.01**	2914	3371	2412
**>10 and <14 Hours**	**q<0.1**	573	646	370
	**q<0.05**	435	424	260
	**q<0.01**	229	156	107
**>7 and <9 Hours**	**q<0.1**	256	434	129
	**q<0.05**	141	197	63
	**q<0.01**	48	30	12

COSOPT and Fisher's G-test were performed to identify rhythmic transcripts. The number of cycling genes detected by each algorithm is shown at increasingly stringent false-discovery rates.

Strikingly, there were two additional clusters of genes in liver cycling with a frequency two or three times faster than the circadian clock, a second and third harmonic of circadian gene expression (Figure 1A). We identified 260 transcripts that oscillate with a period length of approximately 12 h, and 63 transcripts with a period of approximately 8 h at a false-discovery rate of <0.05. Traces from the microarray expression data show examples of 24, 12 and 8 h cycling genes (Figure 1B–D). Table 1 summarizes the results of this statistical analysis, while the complete list of all liver cycling genes can be found in Tables S1, S2, S3.

Although 24 h transcriptional rhythms are well characterized, to our knowledge there has been no previous studies that either observe or predict the presence of circadian harmonics. Therefore, we took several steps to validate the results of our microarray studies. First, we tested the possibility that these ultradian rhythms may be variants of a 24 h rhythm. To this end, we re-ran COSOPT on both the 12 h genes (n = 260) and the 8 h genes (n = 63) while restricting the possible period lengths to either circadian or ultradian rhythms (Figure S2). We found that in practically every case ultradian period lengths more successfully fit these data than conventional circadian rhythms (median p-values of 0.001 and 0.002 for 12 h and 8 h datasets, respectively). In contrast, circadian period lengths (i.e. >20 and <28 h) dramatically failed to detect rhythms in these data (median p-values of 0.4 and 1.0 for 12 h and 8 h datasets, respectively).

Second, to verify experimentally the presence of sub-circadian transcriptional rhythms, an independent time course of mouse liver samples was collected and analyzed using quantitative PCR (qPCR). In this experiment, both core clock and sub-circadian genes oscillated with period lengths in agreement with the original microarray study (Figure S3). Typically, 12 h rhythms showed closer agreement between independent experiments than 8 h rhythms, however, in both cases, there is evidence of agreement between microarray and qPCR profiles.

**Figure 3:**
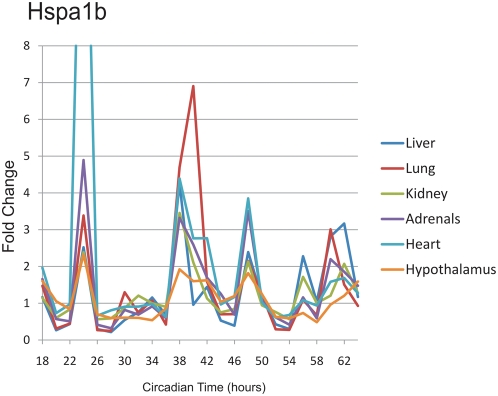
qPCR profiling of Hspa1b reveals 12 h rhythms in multiple tissues. RNA samples from six different tissues were collected at a two-hour resolution between CT18 and CT64. These samples were analyzed using qPCR probes, median normalized, and plotted against CT time. Notably, in every tissue tested, Hspa1b shows four peaks of expression during the 48 h time course; in every case, the phase of these rhythms is invariant between tissues.

Third, 48 h collections were made from a number of different tissues, and qPCR was used to examine the gene expression of a handful of known 12-h cyclers from the liver. One transcript, Hspa1b, showed clear 12 h transcriptional rhythms in every tissue tested (Figure 3), indicating that the presence of circadian harmonics is not restricted to the liver. Strikingly, the phase of Hspa1b rhythms was nearly identical between tissues, suggesting a common underlying mechanism. At the same time, gene expression analysis of additional 12 h genes shows that many transcripts revert to 24 h periodicity in tissues outside the liver (Figure S4), suggesting that 12 h rhythms are driven by both systemic circadian cues and local, tissue-specific factors. Interestingy, the expression patterns of Hspa5 and Armet show considerable similarity across multiple tissue types, reinforcing the possibility that these genes (and thus their rhythms) are driven by systemic cues.

**Figure 4:**
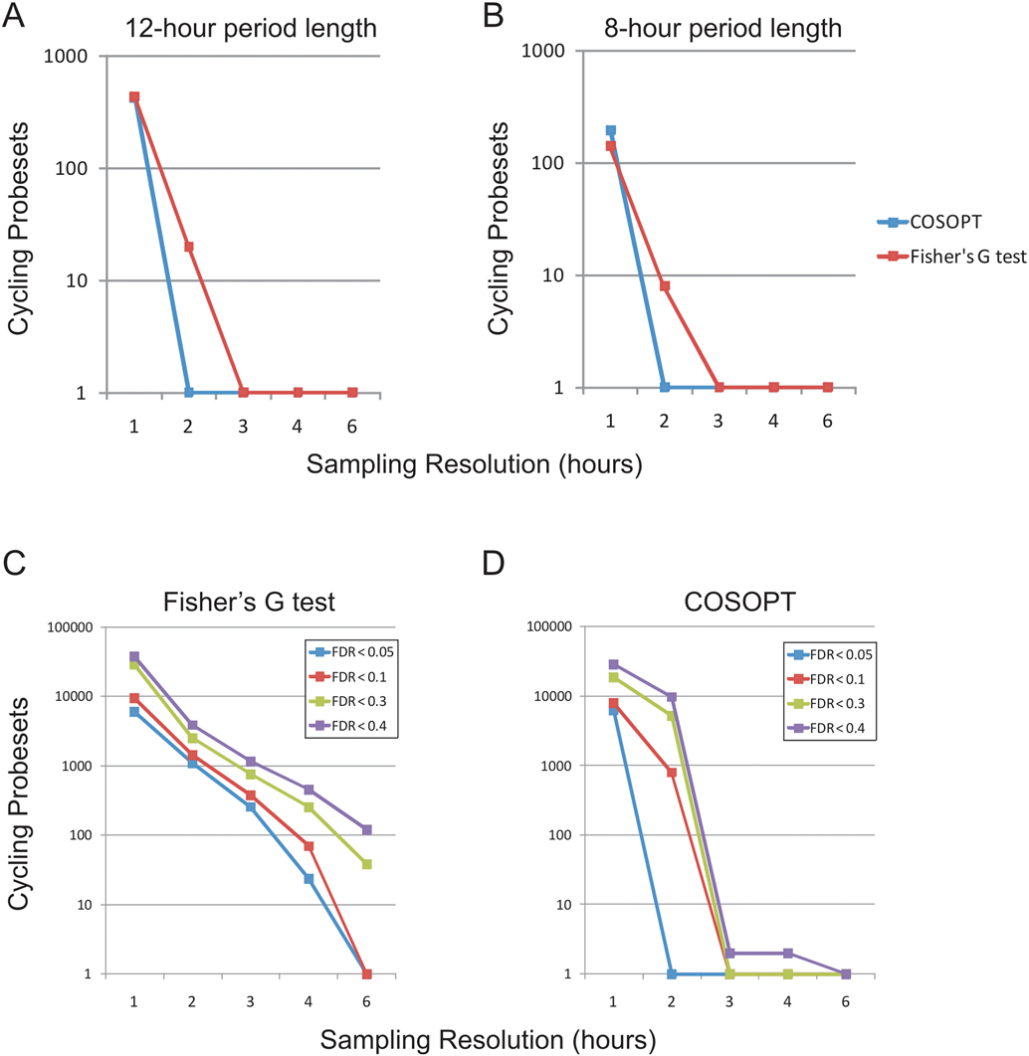
High temporal resolution is required to detect 12 and 8 h rhythms. COSOPT and Fisher's G-test were performed on subsets of the microarray data set to simulate the statistical power of sampling at a 1, 2, 3, 4 or 6 h resolution. The number of rhythmic genes detected at a FDR of <0.05 by either algorithm is plotted against the sampling resolution for 12 h (period >10 and <14) (A) or 8 h genes (period >7 and <9) (B). In each case, one hour sampling resolution is required to optimally detect transcriptional rhythms. Additional simulations revealed that both Fisher's G test (C) and COSOPT (D) detected considerably more rhythmic transcripts of all period lengths when samples were taken at a two-hour resolution or better. At very high FDRs (e.g. <0.4), using single algorithms, a sizable proportion of the genome is found to cycle. This observation underscores the importance of using appropriately low FDRs as well as multiple algorithms to cross-validate cycling genes.

These 12 h rhythms were not seen in NIH3T3 or U2OS cells (Figure 2, Tables S4 and S5), nor a second tissue, the pituitary gland, analyzed in the same fashion [24]. The novelty of this observation can be explained in part by the statistical power of the current study. Simulations reveal that both 12 and 8 h cycling genes are undetectable by conventional 4 h sampling densities (Figure 4A–B). Moreover, both Fisher's G-test and COSOPT were found to be dramatically underpowered when used at sampling densities less than every 2 h (Figure 4C–D). In contrast, by increasing the frequency of time points to every 2 or 1 h, substantial numbers of additional cycling genes can be detected at low false-discovery rates (Figure 4C–D).

**Figure 5:**
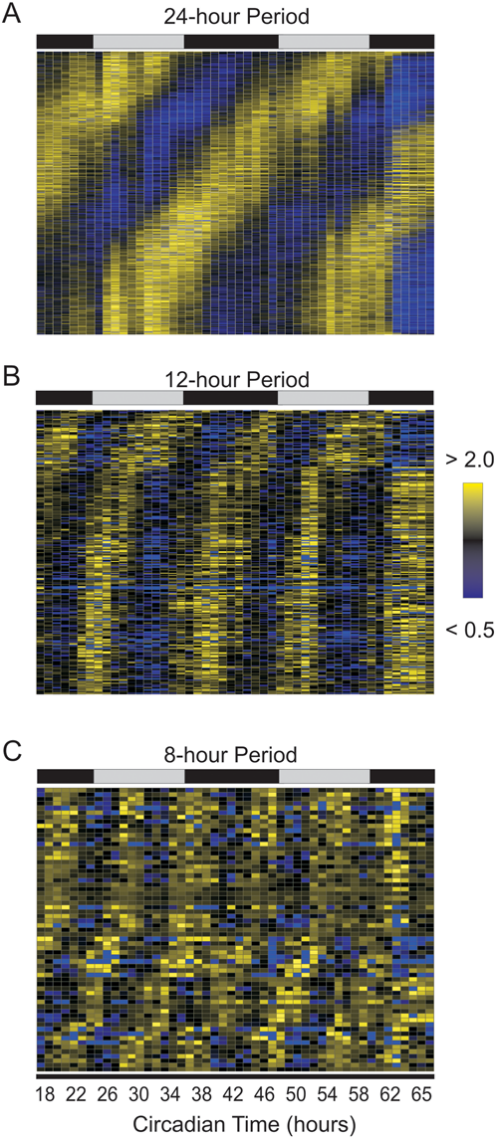
The peaks of 12 h cycling genes correlate with subjective dusk and dawn. Rhythmic transcripts detected by COSOPT and Fisher's G-test at a false-discovery rate of <0.05 were median-normalized and plotted as a heatmap for 24, 12 and 8 h cycling genes (A–C). Bright yellow represents expression 2-fold greater than median levels while bright blue represents expression less than 50 percent of median levels. The time of peak expression of 24 h cycling genes show a roughly equal distribution over the course of a day; in contrast, the peak expression of both 12 h rhythms are biased to specific times each day.

In addition to improving the confidence by which both circadian and sub-circadian genes are identified, a 1 h sampling density increases the precision of phase estimates. At a 4 h resolution, only six different phases can be confidently assigned to circadian genes; in contrast, the current study allows the discrimination of phase differences of as little as 1 h. Consequently, subtle but nonetheless consistent phase differences have been identified between core components of the circadian clock (Figure S5). To extend this result, the expression of all cycling genes was median-normalized and plotted as a heat map (Figure 5). Conventional circadian genes show peak expression levels throughout the day with little bias in their phase (Figure 5A). In contrast, the majority of 12 h genes cluster into a single group with similar phases (Figure 5B). Interestingly, the peak of most 12 h genes coincides with dusk and dawn, suggesting that these genes may anticipate the stress of these daily transitions in light and darkness.

Similarly to transcripts with the circadian rhythm, 12 h rhythmic transcripts are involved in a number of different pathways and processes (Table S6). Ingenuity pathway analysis reveals that a number of 12 h rhythmic genes are integrally involved in regulating cell division and protein processing while 8 h rhythms may be involved in NF-kB signaling and lipid metabolism (Figure S6). Taken as a whole, however, available annotations suggest that sub-circadian rhythms regulate a broad spectrum of cellular physiologies.

Like circadian transcripts, the majority of 12 and 8 h genes in the liver oscillate with between 1.5 and 4-fold amplitude (Figure S7). However, core components of the circadian clock (e.g. *Bmal1* and *Per2*) cycle with exceptionally strong amplitudes, while most 12 and 8 h oscillatory transcripts rarely demonstrate greater than 10-fold amplitudes. In addition, unlike circadian transcripts, many 12 h genes show differences in amplitude between their morning and evening peaks, which suggests the possibility that different physiological signals are responsible for driving the twice-daily peaks of 12 h rhythms (Figure S8).

To test whether 12 h rhythms persist in the cultured cells, we compared their expression profiles in commonly used models of the autonomous circadian clock, NIH3T3 and U2OS cells. Using the same statistical analysis as above, neither 12 nor 8 h rhythms were detected in either cell line and fewer than a dozen (mostly core clock components and first order clock controlled genes) showed clear circadian oscillations (Figure 2, Table S4, S5). This paucity of cycling output genes was not due to poor oscillator function, as clear rhythms in reporter gene expression could be seen in parallel experiments (Figure S1). Most importantly, the RNA expression profiles of core circadian genes showed amplitudes of oscillation in agreement with previous studies of circadian cell lines (Figure 2, online supplemental data) [17],[29],[30]. Although core-clock components oscillated as expected, the amplitude of individual components was dampened relative to their profiles in liver and there was no evidence for sub-circadian rhythms (Figure S9, Tables S4, S5). This result was validated using an independent sample collection and qPCR (Figure S10). When compared to high-resolution circadian profiling of the liver and pituitary [24], these data indicate that NIH3T3 and U2OS cells recapitulate the oscillations of core clock components, but fail to adequately maintain robust circadian transcriptional output seen *in vivo* (Figure S11).

**Figure 6:**
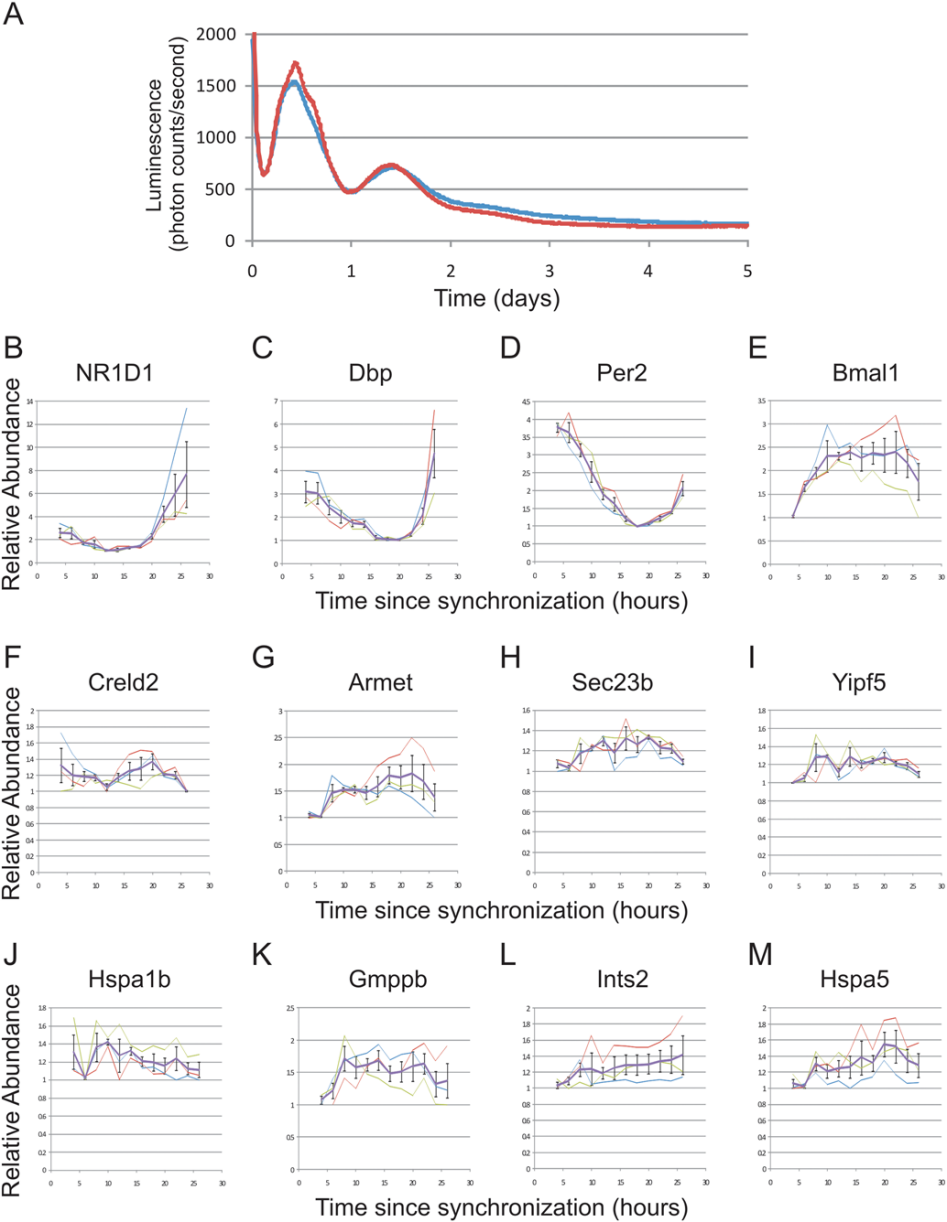
12 h rhythmic transcription is dampened in *ex vivo* hepatocytes. Primary hepatocytes were prepared from *Per2-luciferase* mice and shocked with dexamethasone to synchronize their circadian clocks. Real-time luciferase measurements revealed a circadian oscillation which dampens over the course of three days *in vitro* for two replicates shown in red and blue (A). Starting four h after dexamethasone shock, mRNA samples from these cells were collected every two h for an entire day and quantitative PCR was used to assess the levels of endogenous mRNAs. Core clock genes, including NR1D1, Dbp, Per2 and Bmal1, were rhythmic over the analyzed time points (B–E); however, 12 h genes were either severely dampened (F) or were completely arrhythmic (G–M). Error bars are +/−S.E.M.; thick purple traces represent the average of three replicates, thin traces show the result of each individual replicate.

An obvious caveat to this observation is the possibility that tissue-specific cues may drive sub-circadian oscillations in hepatocytes, but not in fibroblasts or osteosarcoma cells. To examine this, disassociated cultures of primary hepatocytes were prepared from *Per2:Luc* [9] mice and synchronized with dexamethasone. Real-time imaging of luciferase (Figure 6A) as well as qPCR of core clock genes (Figure 6B–E) demonstrated their oscillations with a period of approximately 24 h. The gene expression pattern of Per2 may reflect its role as an immediate early gene; however, the expression patterns of Bmal1, Dbp and Nr1d1 all suggest the presence of an oscillating 24 h clock. Similar to NIH3T3 and U2OS cells, the amplitude of the core clock genes in this system is dampened relative to samples taken from intact liver *in vivo*. However, 12 h oscillations were either severely dampened (Figure 6F) or entirely absent (Figure 6G–M, statistical analysis: Table S7). Combined with the results from NIH3T3 and U2OS cells, these data show dampening of both circadian rhythms and their harmonics in three different isolated cellular models. Given the sensitivity of gene expression assays, it is impossible to distinguish between loss of harmonic oscillations and extremely low amplitude cycling, but for practical purposes, these cellular models are not useful for the study of ultradian rhythms.

**Figure 7:**
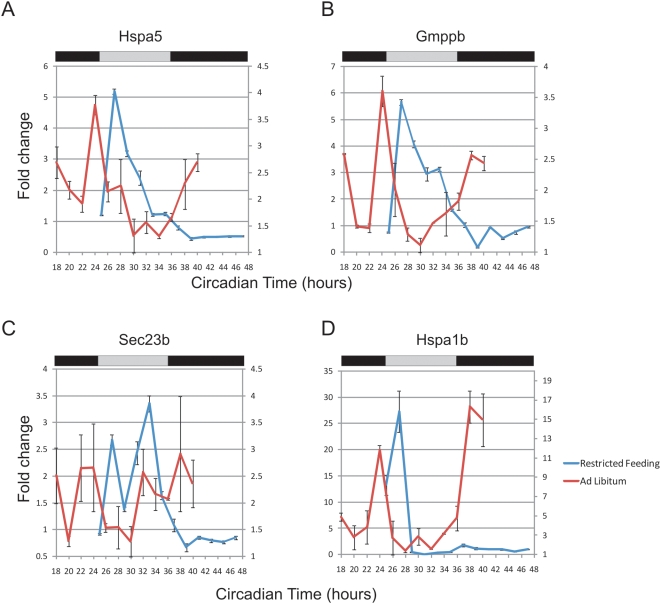
Restricted feeding changes the periodicity of 12 h rhythms. Mice were held in a restricted feeding paradigm (see Methods) and liver samples were collected every 2 h. Quantitative PCR was used to assess the transcriptional profile of eight genes: Hspa5 (A), Gmppb (B), Sec23b (C), Hspa1b (D), as well as Gramd3, Creld2, Gosr2 and Ints2 (data not shown). When compared to samples from ad libitum fed mice (red traces, right axis), restricted feeding samples (blue traces, left axis) showed only a single peak of expression over the course of a complete day (Error bars are +/−S.E.M.).

Food metabolism represents a candidate driver of these cues. To address this, we examined 12 h transcripts in a restricted feeding paradigm. Under normal circumstances, mice feed almost exclusively during the night and generally have a larger meal shortly after lights out [31]. In this restricted feeding design, the availability of food is restricted to an 8 h time window during the subjective day, when mice are normally asleep and not eating. Previous experiments have shown that core clock components in the liver invert their phase by 12 h during restricted feeding [32]. We tested the expression pattern of 12 h genes using quantitative PCR and found that seven of the eight genes dramatically changed their expression patterns in response to restricted feeding, while one transcript became entirely arrhythmic (Figure 7 and data not shown). These genes maintained peak expression at approximately CT26, coinciding with feeding; however, the subjective evening peak was largely absent. Taken as a whole, these data support the hypothesis that at least one component of 12 h rhythms are driven by feeding.

## Discussion

Here we have used genome-scale RNA profiling to identify and compare rhythmic transcripts from mouse liver and two models of the autonomous circadian clock, NIH3T3 cells and U2OS cells, at a 1 h time resolution. To detect rhythmic genes, we have employed a pair of statistical algorithms with different underlying principles to score every transcript for evidence of rhythmicity without bias to period length or amplitude. Our simulations indicate that increasing the sampling resolution of circadian profiling studies dramatically increases the confidence with which cycling genes can be detected and minimizes both false positive and false negative observations (Figure 4). To stimulate use by biologists, these data have been made available to the public by depositing raw data in GEO and using a web-based interface http://bioinf.itmat.upenn.edu/circa/mouse.

It is our hope that this resource will fuel additional investigations into mechanisms of physiological rhythms. For example, these data may be used to identify candidate rhythmic genes which may govern behavioral or physiological rhythms. Alternatively, these data may suggest that a given gene or pathway has a previously unsuspected circadian component to its transcription or mRNA abundance. In either case, the cost of false-positives in our dataset would be considerable in terms of time and resources spent following bad leads.

Therefore, to be most useful to future studies, we have employed the q-value statistic based on the concept of false-discovery rate [27],[28] to estimate the likelihood that a given transcript identified as cycling is in actuality non-rhythmic. q-values for every transcript in this study are available on the web-based interface described above. For the purposes of this manuscript, we have chosen to estimate the total number of cycling genes in each dataset using a q-value threshold of <0.05. In the liver, this confidence level allowed the detection of over 3000 cycling transcripts. Unexpectedly, fewer than a dozen cycling genes were detected using the same statistical paradigm in NIH3T3 and U2OS cells, in contrast with previously published work [17],[29],[30]. We suggest that the increased statistical rigor enabled by higher density profiling has led to both fewer false positives and negatives in detection of oscillating genes. Consistent with this notion, we sampled our data at a 4 h resolution, did not account for multiple testing, and found similar levels of oscillating transcription as reported in previous studies (Table S8). However, when corrected for multiple testing, most of these transcripts are not considered significant at a false-discovery rate <0.05. In other words, they may truly be cycling, but not at that false discovery rate, which allows for only one false positive picked amongst 20 truly cycling transcripts. As the majority of detected cycling genes in these cells are either core clock components or 1^st^ order output genes, we are convinced that these cells will continue to be a fruitful model for studies of circadian clockwork. However, the relative paucity of rhythmic genes in cultured cells is cause for caution regarding studies of circadian output in these systems.

Genetic and epigenetic variations accumulated over many years *in vitro* may account for the loss of robust circadian output in these cell lines. Additionally, the isolation of these cells from circulating cues normally found *in vivo* may contribute to this phenotype. Based on the loss of amplitude of clock oscillations we observed in disassociated hepatocyte cultures (Figure 6), we speculate that *in vitro* techniques to synchronize cultured cells may insufficiently reproduce systemic cues that synchronize and drive rhythmic gene expression *in vivo*. Recently, Schibler and colleagues have shown that the peripheral clock oscillations are necessary for most circadian output [8]. This elegant study, however, does not address the sufficiency of these autonomous cellular models to generate robust rhythmic transcripts. In combination with the results of the Schibler group, we suggest the possibility that robust circadian output in the liver may depend on the combination of an intact peripheral clock as well as circulating, rhythmic cues found in intact animals.

Surprisingly, during the course of this investigation, we discovered second and third harmonics of circadian gene expression in liver and using qPCR subsequently validated 12 h rhythmic transcription in liver as well as in several other peripheral tissues. Several lines of evidence suggest that these rhythms are driven by systemic, circulating cues rather than distinct self-sustained molecular clocks. First, similar to circadian output in NIH3T3 and U2OS cells (Figure 2), 12 h oscillations are dramatically dampened in *ex vivo* hepatocytes (Figure 6), consistent with the possibility that external signals synchronize and/or reinforce these rhythms *in vivo*. Furthermore, systemic cues triggered by restricted feeding substantially change the expression pattern of a subset of these genes by eliminating the evening peak of expression (Figure 7). We speculate that 12 h transcriptional rhythms may be generated by changes in behavior and stress-levels coincident with phase-transitions, and may thus provide an advantage to organisms that need to anticipate dusk and dawn. In this model, two or more physiological rhythms with a 24 h period (e.g. feeding behavior) may integrate to generate ultradian rhythms in peripheral tissues. These cues need not be transcriptional, one could envision a transcriptional rhythm of 24 hours intersecting with an out of phase 24 h RNA degradation rhythm producing apparent 12 h rhythms in transcript levels. Interestingly, a number of proteins involved in mediating endoplasmic reticulum (ER) stress, including hsp70 and the transcription factor XBP1, have been independently shown to oscillate at the protein level with 12 h period lengths (F. Gachon, personal communication). Taken together, these data suggest an attractive hypothesis that feeding behavior and food metabolism may regulate 12 h rhythms via the ER stress machinery.

Our investigations have also shown that at least one gene, Hspa1b, also known as HSP 70-2, a heat shock factor that also regulates many processes including immune system function and metabolism, cycles with a 12 h period in at least six different tissues (Figure 3). These data strongly suggest that ultradian transcriptional rhythms have importance beyond the liver. However, the prevalence of non-24 h rhythms in additional peripheral tissues as well as the extent to which they depend on the tissue-autonomous circadian clock remain open questions and the subject of further investigation. Importantly, these data demonstrate the existence of non-24 h biological rhythms and a screening methodology by which to discover them. Finally, these data emphasize the idea that robust rhythms *in vivo* are a product of interactions between autonomous circadian clocks and systemic cues that are difficult to replicate *in vitro*.

## Materials and Methods

### Circadian Tissue Collection

Collection of liver time points was performed as previously described [14]. Briefly, 6-week-old male C57BL/6J mice (Jackson) were housed in light-tight boxes and entrained to a 12 h light, 12-h dark schedule for one week before being switched to complete darkness. Starting at CT18, 3–5 mice were sacrificed in the dark per time point. Liver samples were quickly excised and snap-frozen in liquid nitrogen. Mice under a restricted feeding regiment were allowed access to food between ZT1 and ZT9. To prevent hoarding of food, the mice were subject to cage changes twice a day, alternating between feeding and fasting cages. Control animals were similarly handled, with the exception that food was present in both cages. All animal experiments were performed with the approval of the Institutional Animal Care and Use Committee.

### Microarray Analysis

Liver and cell samples were homogenized in Trizol (Invitrogen) and RNA was extracted with RNeasy columns using the manufacturer's protocol (Qiagen). RNA expression for the liver and NIH3T3 cells was assayed using Affymetrix Mouse Genome 430 2.0 array and data were extracted using GCRMA implemented in ‘R’. Present/absent calls were made using MAS5 in Expression Console (Affymetrix) for Mouse Genome 430 2.0 arrays; liver arrays had an average of 18,581 present transcripts (41.2%), NIH3T3 arrays had an average of 29,220 present transcripts (64.8%). Samples from U2OS cells were analyzed on Affymetrix Human Gene 1.0 ST arrays and the data was extracted using Expression Console (Affytmetrix). Present/absent calls were made using RMA in Expression Console (Affymetrix) at an exon level; U2OS arrays had an average of 68,169 present transcripts (26.5%). COSOPT and Fisher's G-test were performed as described [24], and the raw data and statistics were complied into an Access database (Microsoft). All .cel files are available from GEO (liver accession = GSE11923, NIH3T3 accession = GSE11922, U2OS accession = GSE13949) and microarray data are available in a web-based interface at http://bioinf.itmat.upenn.edu/circa/mouse/.

### Quantitative PCR

1 µg total RNA was used to generate cDNA with the High Capacity cDNA Archive Kit using the manufacturer's protocol (Applied Biosystems). Quantitative PCR reactions were performed using iTaq PCR mastermix (BioRad) in combination with gene expression assays (Applied Biosystems) on a 7800HT Taqman machine (Applied Biosystems). Importin 8 (Mm01255158_m1) was used as an endogeneous control for all experiments. Primer and probe information is available from the manufacturer's webpage: Bmal:Mm00500226_m1, Dbp:Mm00497539_m1, Gramd3: Mm00509320_m1, Gmppb:Mm00626032_g1, Gosr2:Mm00444711_m1, Hsap5:Mm00517691_m1, Hspa1b: Mm03038954_s1, Sec23b:Mm00444887_m1, Ints2:Mm00660825_m1, Yipf5:Mm00834912_g1, Creld2:Mm00513021_m1 (Applied Biosystems). All data were analyzed using RQ manager v1.2 (Applied Biosystems).

### Cell Culture

NIH3T3 cells (ATCC) were grown to confluence and synchronized with 10 µM forskolin (Sigma). U2OS cells were grown to confluence and schocked with 0.1 µM dexamethasone (Sigma). Transfections were performed with Fugene HD using the manufacturer's protocol (Roche). Primary hepatocytes were extracted from Per2-luciferase mice [9] as previously described [33], cultured on collagen coated plates (BD Biosciences) in DMEM (Gibco) supplemented with 10% FBS (Hyclone) and synchronized with 0.1 µM dexamethasone (Sigma) after 48 h *in vitro*. Cells were homogenized in Trizol (Invitrogen) and snap frozen in liquid nitrogen at the indicated time points. For lumicycle analysis, cells were cultured in DMEM supplemented with 10% FBS (Hyclone), 10 mM HEPES (Gibco), and 0.1 mM Luciferin, sealed in 35 mm tissue culture dishes and analyzed using a Lumicycle (Actimetrics).

## Supporting Information

Figure S1 Synchronization of NIH3T3 and U2OS cells. NIH3T3 (A) and U2OS cells (B) transfected with a *Bmal1:luciferase* reporter gene (transiently for NIH3T3 cells and stably for U2OS cells) were grown to confluence. Background signal was de-trended using LumiCycle software (Actimetircs) and real-time luciferase measurements revealed a strongly oscillating circadian clock which dampens over the course of three days in vitro. Two replicates shown in red and blue demonstrate the reproducibility of phase and period length for replicate cultures synchronized in parallel. Note that the phase difference between NIH3T3 (A) and U2OS (B) cells is consistent with the RNA profiling shown in Figure 2.
(0.69 MB TIF)

Figure S2 24 h rhythms fail to adequately fit either 12 or 8 h cycling transcripts. In order to test whether 12 and 8 h genes are variants of the 24 h rhythm, COSOPT was used to measure the quality of fit between these genes and cosine curves of different period lengths. Using circadian period lengths (i.e., >20 and <28 h), we found the median p-value of 0.401 for 12 h genes (A) and 1.00 for 8 h genes (B). In contrast, shorter rhythms (>10 and <14 h for 12 h genes and >7 and <9 for 8 h genes) closely fit these data (median p-values of 0.001 and 0.002, respectively). Note in particular the logarithmic scale of the y-axis in both panels.
(0.20 MB TIF)

Figure S3 Quantitative PCR validation of 12 h rhythmic transcription. A second collection of liver samples was performed and qPCR was used to assess the levels of endogenous mRNA. Blue traces represent microarray profiles from the original tissue collection and were plotted on the left axis; red traces represent fold changes observed in qPCR from the second tissue collection and were plotted on the right axis. Both core clock genes (A, B), 12 h genes (C–H), and 8 h genes (I–J) showed a close correlation between experiments. For additional quantitative PCR validation also see Figure 7A–D.
(1.41 MB TIF)

Figure S4 A subset of 12 h genes from the liver revert to 24 h periodicity in different tissues. qPCR analysis was used to assess the RNA profile in multiple tissues of two genes, Hspa5 (A, C, E, G, I) and Armet (B, D, F, H, J), which cycle with 12 h rhythms in the liver. Although these genes do not show 12 h periodicity outside of the liver (unlike Hspa1b, Figure 3), in several tissues they show robust circadian rhythms (e.g., within the Kidney and Heart). The original liver microarray traces for Hspa5 (K) and Armet (L) (previously shown in Figure S3) have been reprinted here to ease comparisons between experiments.
(0.89 MB TIF)

Figure S5 Relative phasing of core clock genes in liver, pituitary and NIH3T3 and U2OS cells. The timing of peak-expression of core clock genes in the liver (A), pituitary (B), NIH3T3 cells (C), and U2OS cells (D) was estimated by visual inspection and plotted on a circular phase map.
(5.48 MB TIF)

Figure S6 Ingenuity pathway analysis of subcircadian genes. Rhythmic genes identified by COSOPT and Fisher's G-test at a false-discovery rate of <0.05 were analyzed using Ingenuity pathway analysis. The path designer tool was used to identify networks of rhythmic genes involved in cell division and cancer (A), protein secretion/ER stress response (B), NF-kB signaling (C) and lipid metabolism (D). Genes in red cycle with 24 h periods, genes in yellow cycle with 12 h periods, and genes in green cycle with 8 h periods.
(1.52 MB TIF)

Figure S7 Circadian transcripts oscillate with modestly higher amplitudes than either 12 or 8 h genes. The amplitude of cycling transcripts was estimated by calculating the peak to trough ratio ( = percentile[0.95 , x]/percentile[0.05 , x]) and plotted as a histogram. For 24, 12, and 8 h genes, the majority of cycling transcripts had amplitudes less than 4-fold (A–C); however, circadian transcripts showed a significantly larger proportion of genes with amplitudes greater than 10-fold (A).
(0.38 MB TIF)

Figure S8 Examples of ‘harmonics’ in 12 h genes. Microarray intensity is plotted against CT time for three genes which show ‘harmonics’ of circadian gene expression, Hsap1b (A), Dnaja1 (B), and Dsc2 (C).
(0.56 MB TIF)

Figure S9 Amplitude comparison between liver and NIH3T3 cells. The amplitude of core clock genes was estimated by calculating the peak to trough ratio ( = percentile[0.95 , x]/percentile[0.05 , x]) and graphed alongside the amplitudes of the same genes in the liver. The differences in amplitude we observed were independent of microarray intensity between experiments as indicated by a comparison of the coefficient of variance (standard deviation/mean) for each probe (data not shown).
(0.21 MB TIF)

Figure S10 12 h genes do not cycle in NIH3T3 cells. To validate the microarray profiling of NIH3T3 cells, a second time course of cycling 3T3 cells was collected every 2 h for 48 h. Quantitative PCR was used to measure the fold change of endogenous RNA which was plotted against CT time. Core circadian clock genes including Per 2 (A) and NR1D1 (B) oscillate with periods of approximately 24 h. In contrast, genes with 12-hour periods in the liver are arrhythmic (C–H).
(0.68 MB TIF)

Figure S11 Comparison of Liver, Pituitary NIH3T3 and U2OS datasets. High temporal resolution profiling has been performed on samples from the liver, pituitary [24], U2OS and NIH3T3 cells. Cycling transcripts were detected in the liver and pituitary at a false-discovery rate of <0.05; rhythmic transcripts in U2OS and NIH3T3 cells were identified at a false-discovery rate of <0.1. The number of cycling genes common to each group was plotted as a Venn diagram. In (B), the number off cycling genes common to NIH3T3 cells (in black) and U2OS cells (in red) were plotted as a Venn diagram.
(0.75 MB TIF)

Table S1 24 h cycling genes. Cycling genes were identified which had false-discovery rates less than 0.05 in both COSOPT and Fisher's G-test, as well as a COSOPT period length greater than 20 h and less than 30 h.
(0.88 MB XLS)

Table S2 12 h cycling genes. Cycling genes were identified which had false-discovery rates less than 0.05 in both COSOPT and Fisher's G-test, as well as a COSOPT period length greater than 10 h and less than 14 h.
(0.08 MB XLS)

Table S3 8 h cycling genes. Cycling genes were identified which had false-discovery rates less than 0.05 in both COSOPT and Fisher's G-test, as well as a COSOPT period length greater than 7 h and less than 9 h.
(0.03 MB XLS)

Table S4 NIH3T3 cycling genes. Cycling genes were identified which had false-discovery rates less than 0.1 by COSOPT as well as a COSOPT period length greater than 20 h and less than 30 h.
(0.02 MB XLS)

Table S5 U2OS cycling genes. Cycling genes were identified which had false-discovery rates less than 0.1 by COSOPT as well as a COSOPT period length greater than 20 h and less than 30 h.
(0.02 MB XLS)

Table S6 GO annotation of 12 h genes. Cycling genes were identified which had false-discovery rates less than 0.05 in both COSOPT and Fisher's G-test, as well as a COSOPT period length greater than 10 h and less than 14 h. These genes were analyzed using Spotfire DecisionSite to identify over-represented GO annotation classes.
(0.03 MB XLS)

Table S7 Ex vivo hepatocyte time course statistics. Primary hepatocytes were prepared from Per2-luciferase mice and shocked with dexamethasone to synchronize their circadian clocks. Starting four hours after dexamethasone shock, mRNA samples from these cells were collected every two hours for an entire day and quantitative PCR was used to assess the levels of endogenous mRNAs. Fisher's G-test and COSOPT were used to assess the likelihood that these traces were oscillating and estimate their period length.
(0.02 MB XLS)

Table S8 Sampling the NIH3T3 and U2OS datasets at 4 h resolution yields similar results to previous profiling studies of circadian cell lines. In order to determine the importance of high resolution temporal sampling and false discovery-rate corrections, both the NIH3T3 dataset and the U2OS dataset were analyzed by COSOPT using one quarter of the time points to simulate 4 h sampling. At a p-value cutoff of <0.05, thousands of transcripts representing 5–10% of the genome were declared rhythmic by COSOPT, consistent with the results of previous studies. Increasing the sampling resolution to once every hour dramatically increased the number of genes with p-values<0.05. However, in both sampling conditions, very few of these genes demonstrated q-values<0.05, suggesting that the actual number of cycling transcripts in circadian cell lines is considerably lower than previously thought.
(0.02 MB XLS)

## References

[1] Curtis AM, Fitzgerald GA (2006). Central and peripheral clocks in cardiovascular and metabolic function.. Ann Med.

[2] Hastings MH, Reddy AB, Maywood ES (2003). A clockwork web: circadian timing in brain and periphery, in health and disease.. Nat Rev Neurosci.

[3] Klerman EB (2005). Clinical aspects of human circadian rhythms.. J Biol Rhythms.

[4] Levi F, Schibler U (2007). Circadian rhythms: mechanisms and therapeutic implications.. Annu Rev Pharmacol Toxicol.

[5] Halberg F, Cornelissen G, Ulmer W, Blank M, Hrushesky W (2006). Cancer chronomics III. Chronomics for cancer, aging, melatonin and experimental therapeutics researchers.. J Exp Ther Oncol.

[6] Ko CH, Takahashi JS (2006). Molecular components of the mammalian circadian clock.. Hum Mol Genet.

[7] Stratmann M, Schibler U (2006). Properties, entrainment, and physiological functions of mammalian peripheral oscillators.. J Biol Rhythms.

[8] Kornmann B, Schaad O, Bujard H, Takahashi JS, Schibler U (2007). System-driven and oscillator-dependent circadian transcription in mice with a conditionally active liver clock.. PLoS Biol.

[9] Yoo SH, Yamazaki S, Lowrey PL, Shimomura K, Ko CH (2004). PERIOD2::LUCIFERASE real-time reporting of circadian dynamics reveals persistent circadian oscillations in mouse peripheral tissues.. Proc Natl Acad Sci U S A.

[10] Schibler U, Sassone-Corsi P (2002). A web of circadian pacemakers.. Cell.

[11] Storch KF, Paz C, Signorovitch J, Raviola E, Pawlyk B (2007). Intrinsic circadian clock of the mammalian retina: importance for retinal processing of visual information.. Cell.

[12] Storch KF, Lipan O, Leykin I, Viswanathan N, Davis FC (2002). Extensive and divergent circadian gene expression in liver and heart.. Nature.

[13] Harmer SL, Hogenesch JB, Straume M, Chang HS, Han B (2000). Orchestrated transcription of key pathways in Arabidopsis by the circadian clock.. Science.

[14] Panda S, Antoch MP, Miller BH, Su AI, Schook AB (2002). Coordinated transcription of key pathways in the mouse by the circadian clock.. Cell.

[15] Ueda HR, Matsumoto A, Kawamura M, Iino M, Tanimura T (2002). Genome-wide transcriptional orchestration of circadian rhythms in Drosophila.. J Biol Chem.

[16] Lin Y, Han M, Shimada B, Wang L, Gibler TM (2002). Influence of the period-dependent circadian clock on diurnal, circadian, and aperiodic gene expression in Drosophila melanogaster.. Proc Natl Acad Sci U S A.

[17] Duffield GE, Best JD, Meurers BH, Bittner A, Loros JJ (2002). Circadian programs of transcriptional activation, signaling, and protein turnover revealed by microarray analysis of mammalian cells.. Curr Biol.

[18] Ceriani MF, Hogenesch JB, Yanovsky M, Panda S, Straume M (2002). Genome-wide expression analysis in Drosophila reveals genes controlling circadian behavior.. J Neurosci.

[19] Akhtar RA, Reddy AB, Maywood ES, Clayton JD, King VM (2002). Circadian cycling of the mouse liver transcriptome, as revealed by cDNA microarray, is driven by the suprachiasmatic nucleus.. Curr Biol.

[20] McDonald MJ, Rosbash M (2001). Microarray analysis and organization of circadian gene expression in Drosophila.. Cell.

[21] Claridge-Chang A, Wijnen H, Naef F, Boothroyd C, Rajewsky N (2001). Circadian regulation of gene expression systems in the Drosophila head.. Neuron.

[22] Nagoshi E, Saini C, Bauer C, Laroche T, Naef F (2004). Circadian gene expression in individual fibroblasts: cell-autonomous and self-sustained oscillators pass time to daughter cells.. Cell.

[23] Baggs JE, Price TS, DiTacchio L, Panda S, FitzGerald GA (2009). Network Features of the Mammalian Circadian Clock.. PLoS Biology.

[24] Hughes M, Deharo L, Pulivarthy SR, Gu J, Hayes K (2007). High-resolution Time Course Analysis of Gene Expression from Pituitary.. Cold Spring Harb Symp Quant Biol.

[25] Straume M (2004). DNA microarray time series analysis: automated statistical assessment of circadian rhythms in gene expression patterning.. Methods Enzymol.

[26] Wichert S, Fokianos K, Strimmer K (2004). Identifying periodically expressed transcripts in microarray time series data.. Bioinformatics.

[27] Storey JD, Tibshirani R (2003). Statistical significance for genomewide studies.. Proc Natl Acad Sci U S A.

[28] Storey JD, Xiao W, Leek JT, Tompkins RG, Davis RW (2005). Significance analysis of time course microarray experiments.. Proc Natl Acad Sci U S A.

[29] Grundschober C, Delaunay F, Puhlhofer A, Triqueneaux G, Laudet V (2001). Circadian regulation of diverse gene products revealed by mRNA expression profiling of synchronized fibroblasts.. J Biol Chem.

[30] Menger GJ, Allen GC, Neuendorff N, Nahm SS, Thomas TL (2007). Circadian profiling of the transcriptome in NIH/3T3 fibroblasts: comparison with rhythmic gene expression in SCN2.2 cells and the rat SCN.. Physiol Genomics.

[31] Feillet CA, Ripperger JA, Magnone MC, Dulloo A, Albrecht U (2006). Lack of food anticipation in Per2 mutant mice.. Curr Biol.

[32] Stokkan KA, Yamazaki S, Tei H, Sakaki Y, Menaker M (2001). Entrainment of the circadian clock in the liver by feeding.. Science.

[33] Klingmuller U, Bauer A, Bohl S, Nickel PJ, Breitkopf K (2006). Primary mouse hepatocytes for systems biology approaches: a standardized in vitro system for modelling of signal transduction pathways.. Syst Biol (Stevenage).

