# Supplementary figures and images for: Harmonics of Circadian Gene Transcription in Mammals
Source: PLoS Genet. 2009 Apr 3;5(4):e1000442. doi: 10.1371/journal.pgen.1000442 (PMC2654964; doi:10.1371/journal.pgen.1000442)

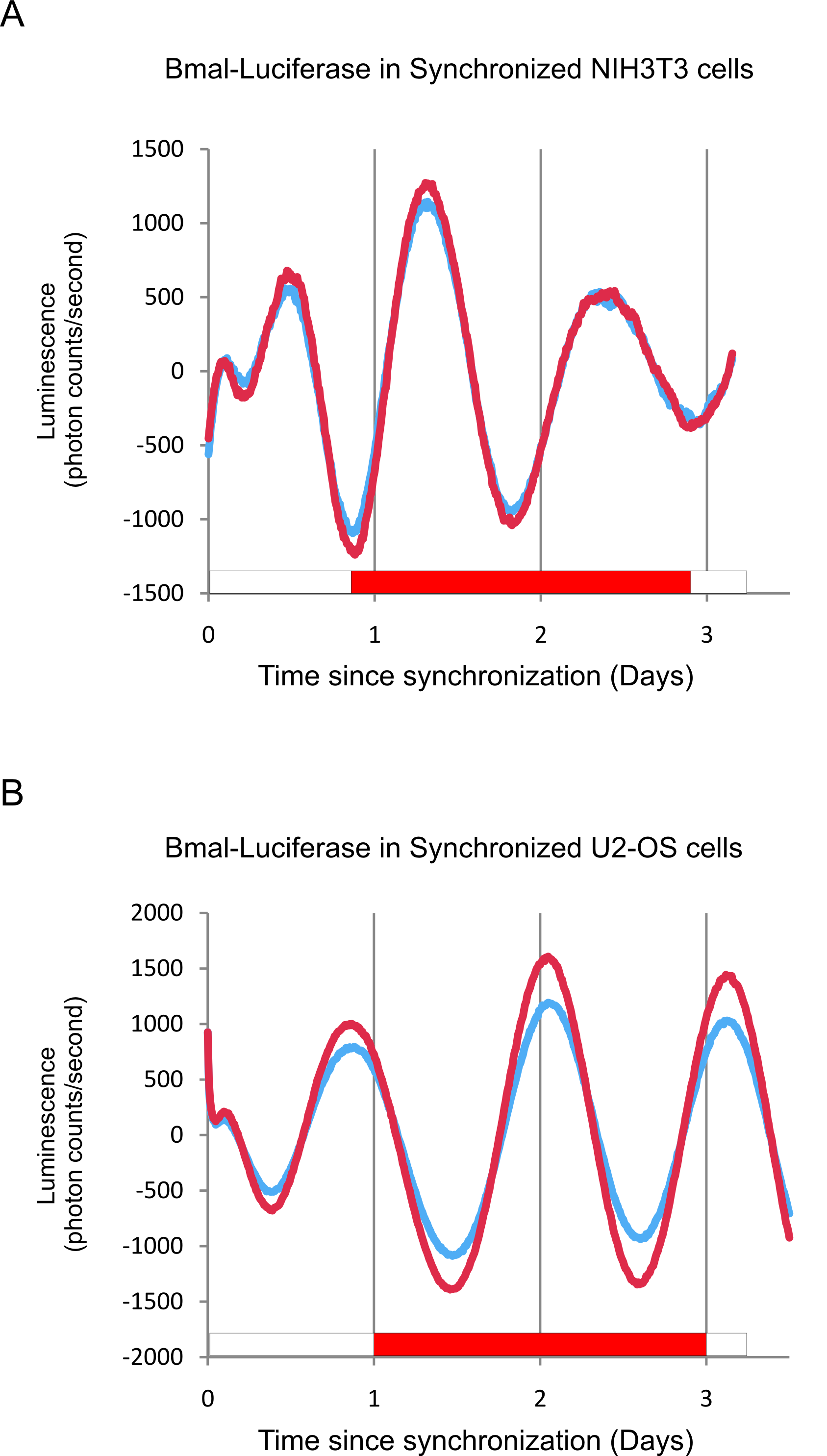

Supplement: Figure S1 — Synchronization of NIH3T3 and U2OS cells. NIH3T3 (A) and U2OS cells (B) transfected with a Bmal1:luciferase reporter gene (transiently for NIH3T3 cells and stably for U2OS cells) were grown to confluence. Background signal was de-trended using LumiCycle software (Actimetircs) and real-time luciferase measurements revealed a strongly oscillating circadian clock which dampens over the course of three days in vitro. Two replicates shown in red and blue demonstrate the reproducibility of phase and period length for replicate cultures synchronized in parallel. Note that the phase difference between NIH3T3 (A) and U2OS (B) cells is consistent with the RNA profiling shown in Figure 2. (0.69 MB TIF) [file pgen.1000442.s001.tif]

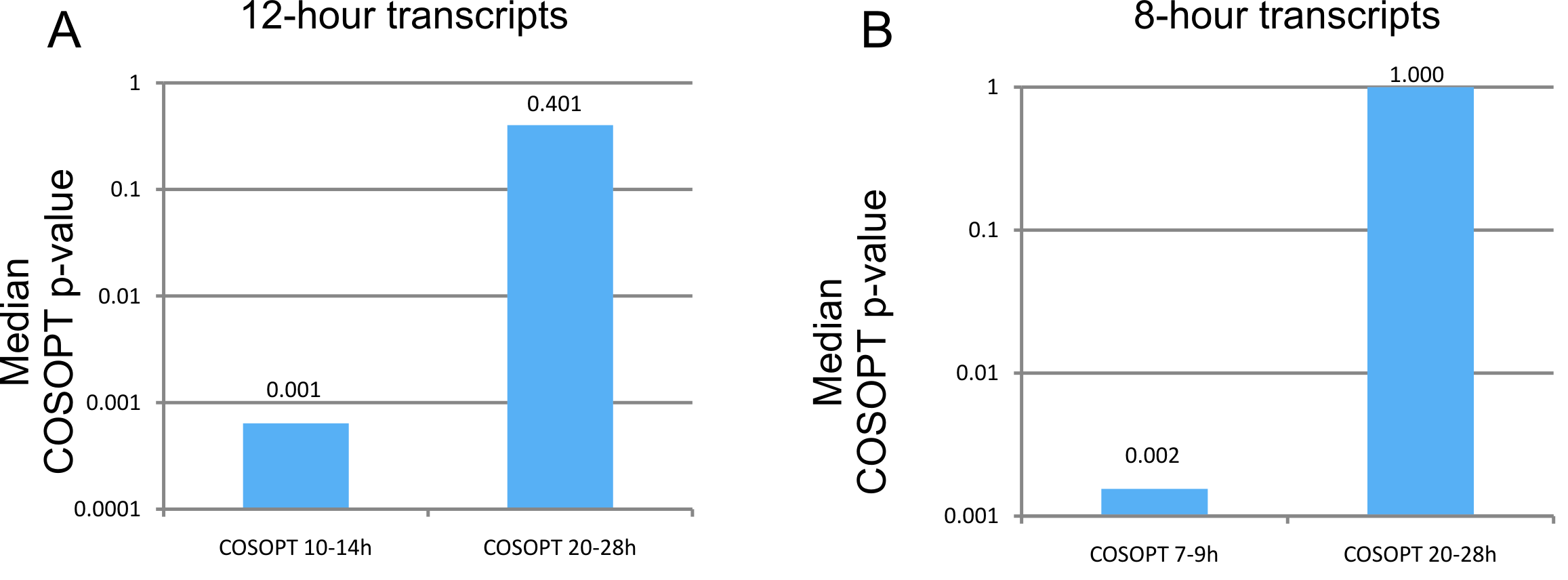

Supplement: Figure S2 — 24 h rhythms fail to adequately fit either 12 or 8 h cycling transcripts. In order to test whether 12 and 8 h genes are variants of the 24 h rhythm, COSOPT was used to measure the quality of fit between these genes and cosine curves of different period lengths. Using circadian period lengths (i.e., >20 and <28 h), we found the median p-value of 0.401 for 12 h genes (A) and 1.00 for 8 h genes (B). In contrast, shorter rhythms (>10 and <14 h for 12 h genes and >7 and <9 for 8 h genes) closely fit these data (median p-values of 0.001 and 0.002, respectively). Note in particular the logarithmic scale of the y-axis in both panels. (0.20 MB TIF) [file pgen.1000442.s002.tif]

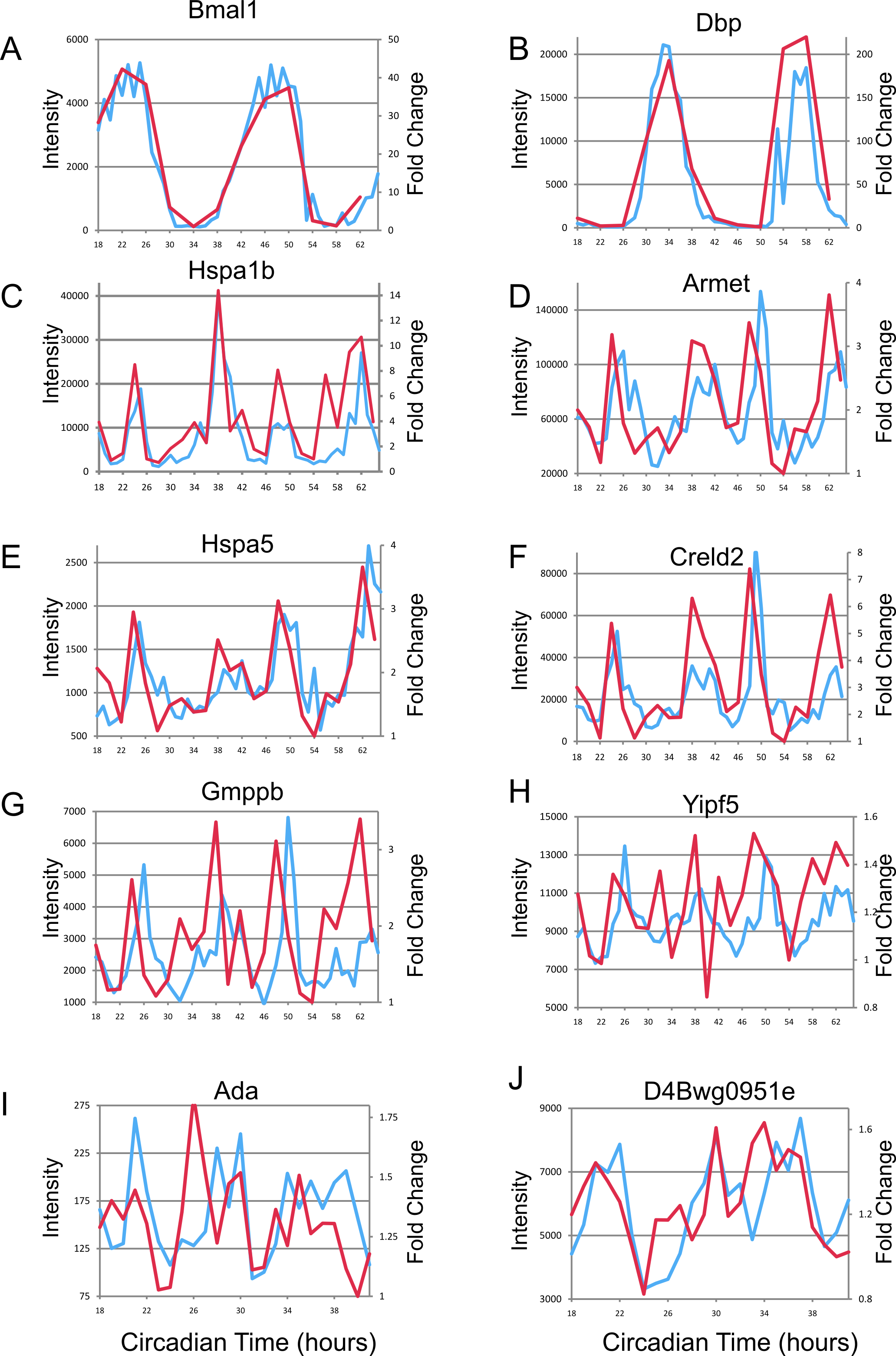

Supplement: Figure S3 — Quantitative PCR validation of 12 h rhythmic transcription. A second collection of liver samples was performed and qPCR was used to assess the levels of endogenous mRNA. Blue traces represent microarray profiles from the original tissue collection and were plotted on the left axis; red traces represent fold changes observed in qPCR from the second tissue collection and were plotted on the right axis. Both core clock genes (A, B), 12 h genes (C–H), and 8 h genes (I–J) showed a close correlation between experiments. For additional quantitative PCR validation also see Figure 7A–D. (1.41 MB TIF) [file pgen.1000442.s003.tif]

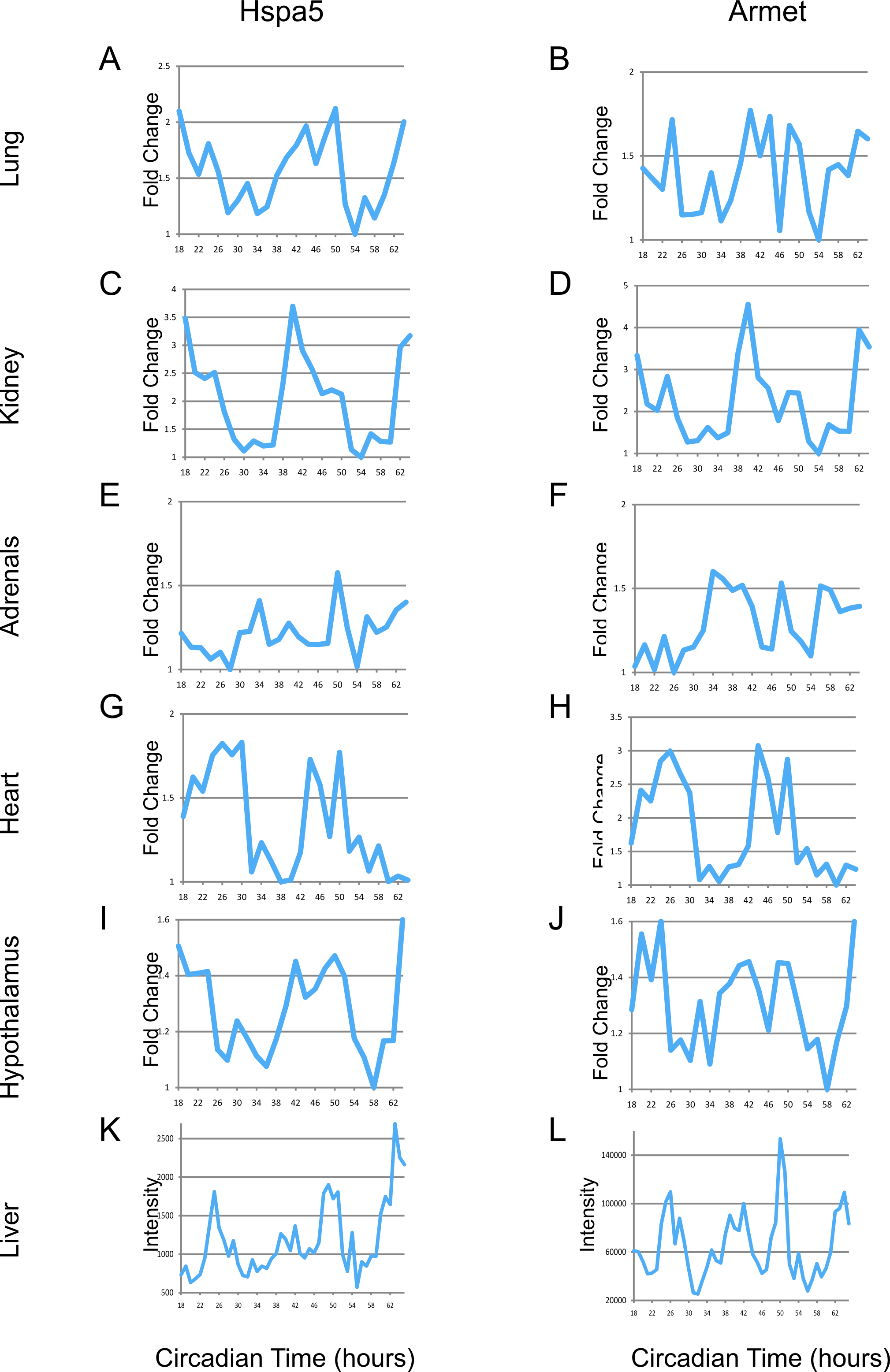

Supplement: Figure S4 — A subset of 12 h genes from the liver revert to 24 h periodicity in different tissues. qPCR analysis was used to assess the RNA profile in multiple tissues of two genes, Hspa5 (A, C, E, G, I) and Armet (B, D, F, H, J), which cycle with 12 h rhythms in the liver. Although these genes do not show 12 h periodicity outside of the liver (unlike Hspa1b, Figure 3), in several tissues they show robust circadian rhythms (e.g., within the Kidney and Heart). The original liver microarray traces for Hspa5 (K) and Armet (L) (previously shown in Figure S3) have been reprinted here to ease comparisons between experiments. (0.89 MB TIF) [file pgen.1000442.s004.tif]

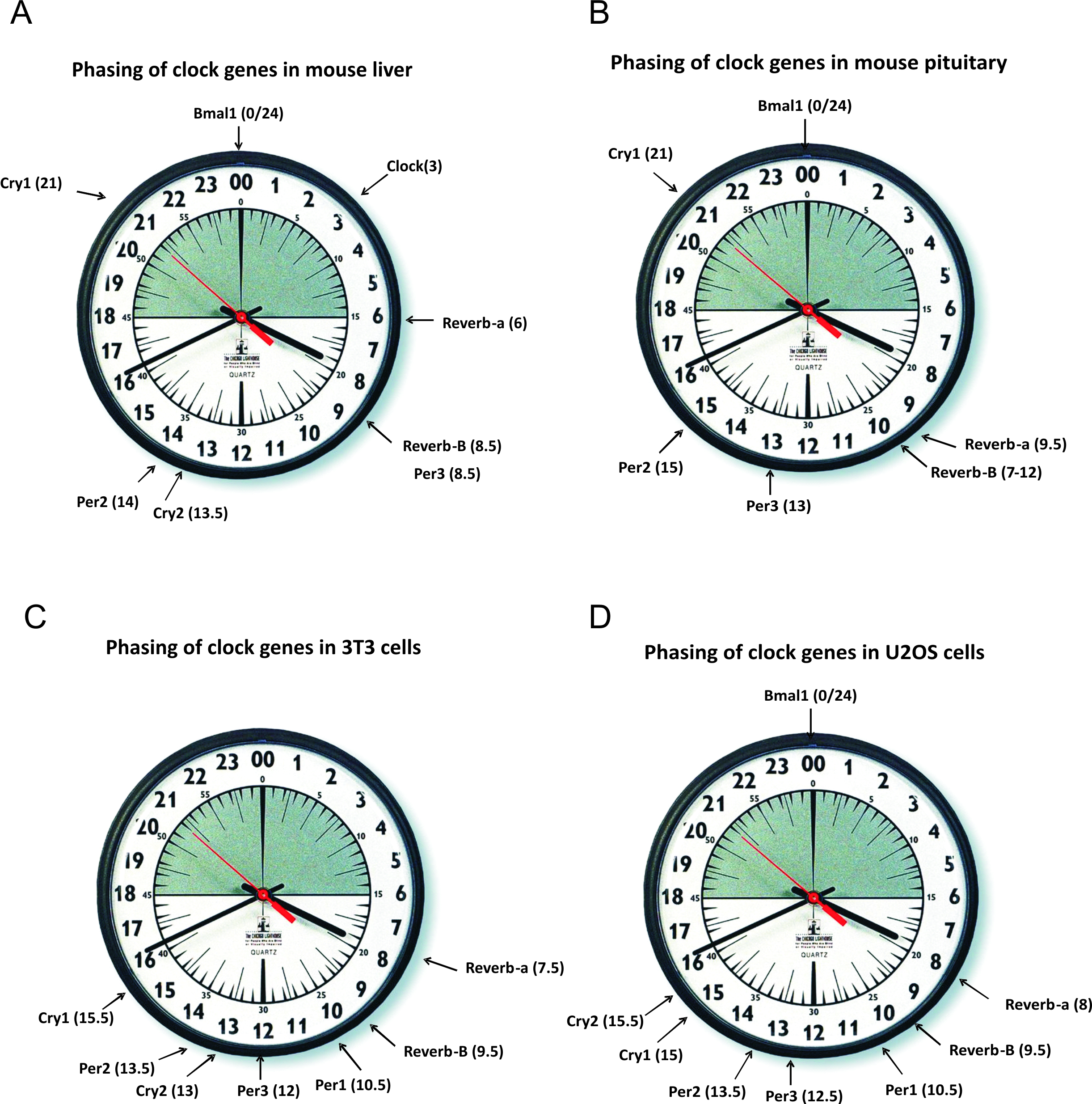

Supplement: Figure S5 — Relative phasing of core clock genes in liver, pituitary and NIH3T3 and U2OS cells. The timing of peak-expression of core clock genes in the liver (A), pituitary (B), NIH3T3 cells (C), and U2OS cells (D) was estimated by visual inspection and plotted on a circular phase map. (5.48 MB TIF) [file pgen.1000442.s005.tif]

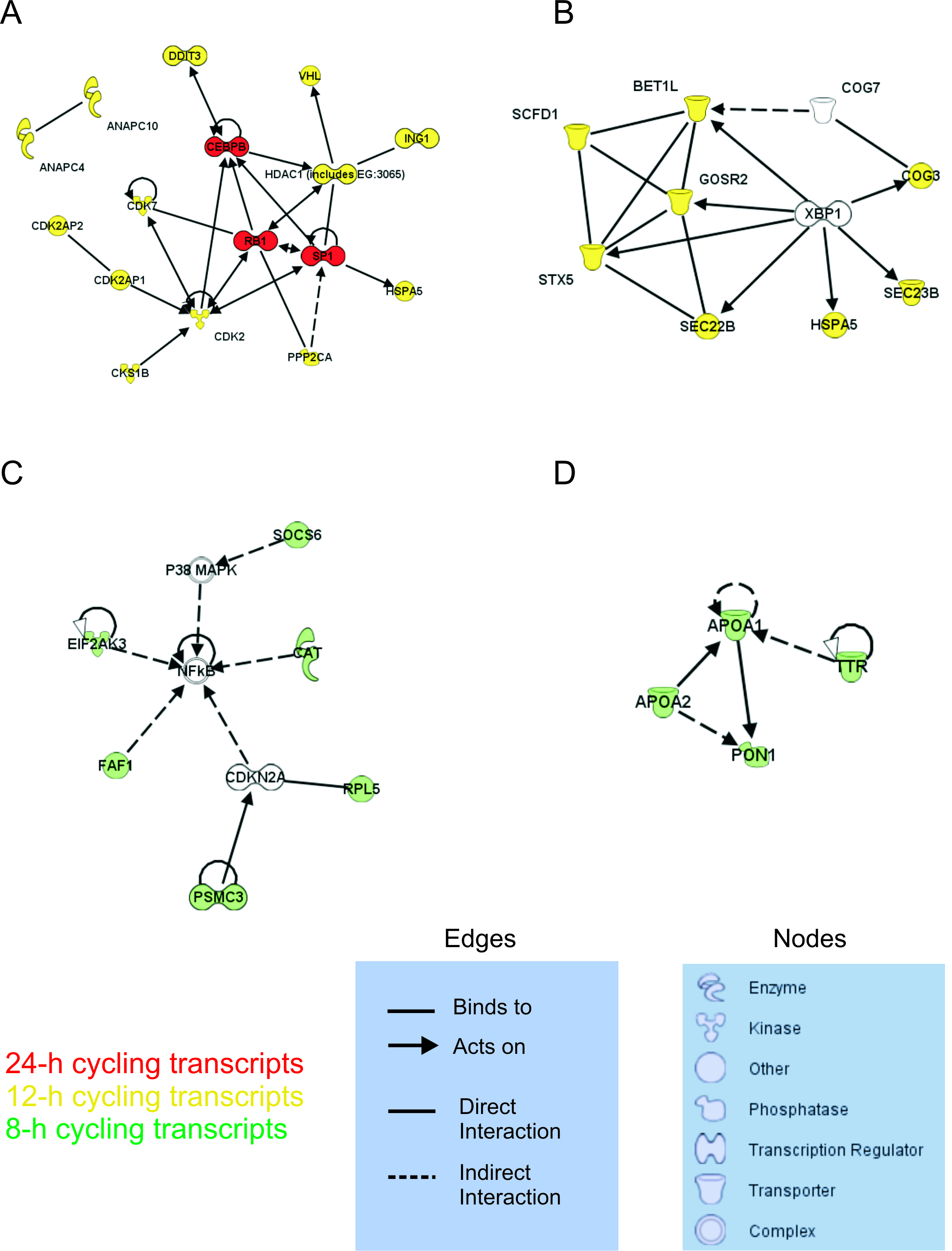

Supplement: Figure S6 — Ingenuity pathway analysis of subcircadian genes. Rhythmic genes identified by COSOPT and Fisher's G-test at a false-discovery rate of <0.05 were analyzed using Ingenuity pathway analysis. The path designer tool was used to identify networks of rhythmic genes involved in cell division and cancer (A), protein secretion/ER stress response (B), NF-kB signaling (C) and lipid metabolism (D). Genes in red cycle with 24 h periods, genes in yellow cycle with 12 h periods, and genes in green cycle with 8 h periods. (1.52 MB TIF) [file pgen.1000442.s006.tif]

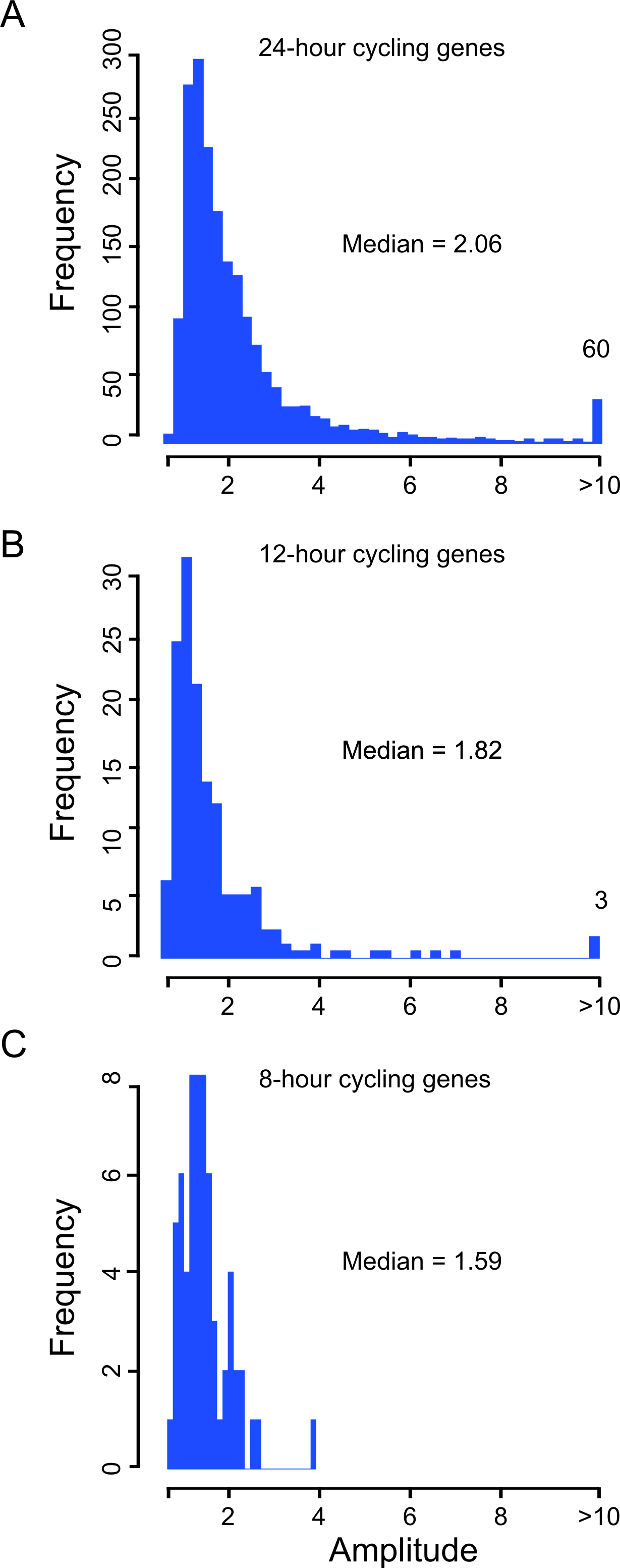

Supplement: Figure S7 — Circadian transcripts oscillate with modestly higher amplitudes than either 12 or 8 h genes. The amplitude of cycling transcripts was estimated by calculating the peak to trough ratio ( = percentile[0.95 , x]/percentile[0.05 , x]) and plotted as a histogram. For 24, 12, and 8 h genes, the majority of cycling transcripts had amplitudes less than 4-fold (A–C); however, circadian transcripts showed a significantly larger proportion of genes with amplitudes greater than 10-fold (A). (0.38 MB TIF) [file pgen.1000442.s007.tif]

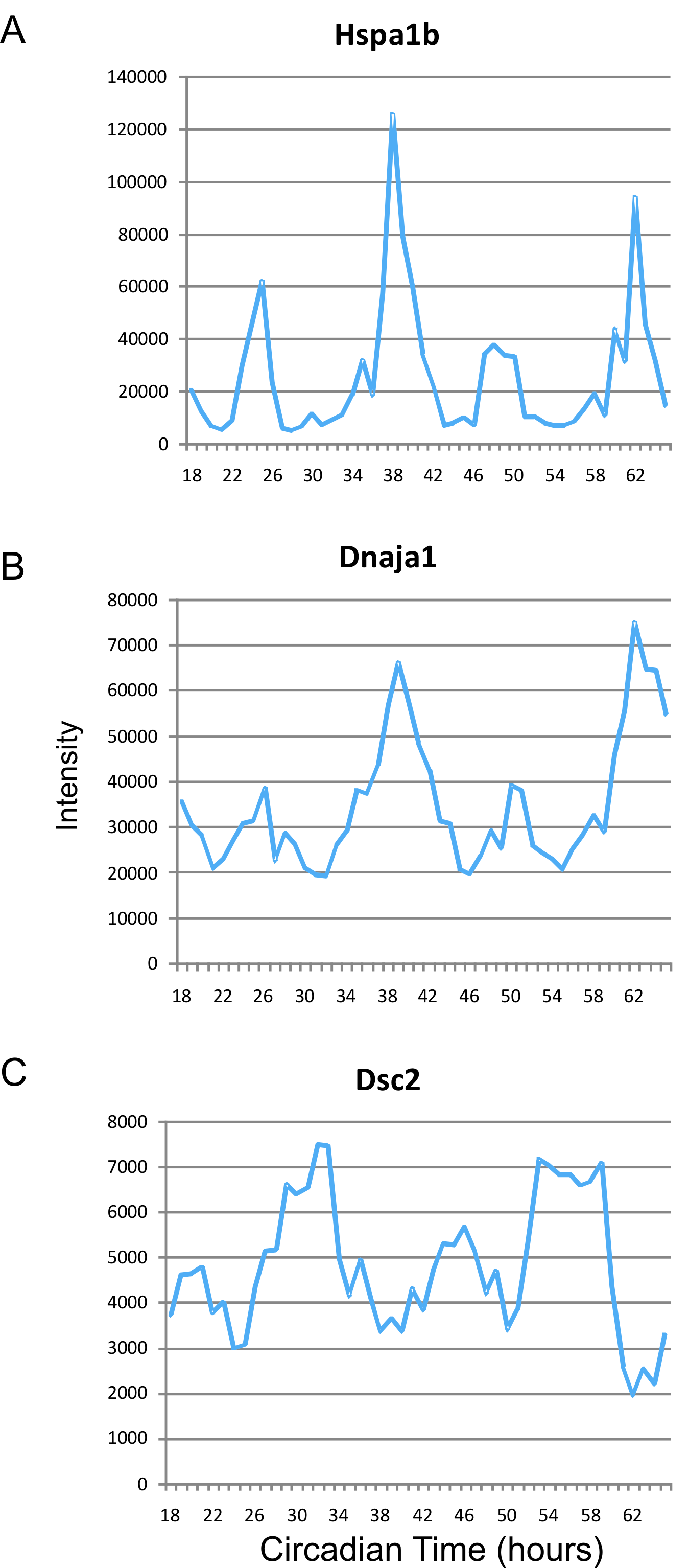

Supplement: Figure S8 — Examples of ‘harmonics’ in 12 h genes. Microarray intensity is plotted against CT time for three genes which show ‘harmonics’ of circadian gene expression, Hsap1b (A), Dnaja1 (B), and Dsc2 (C). (0.56 MB TIF) [file pgen.1000442.s008.tif]

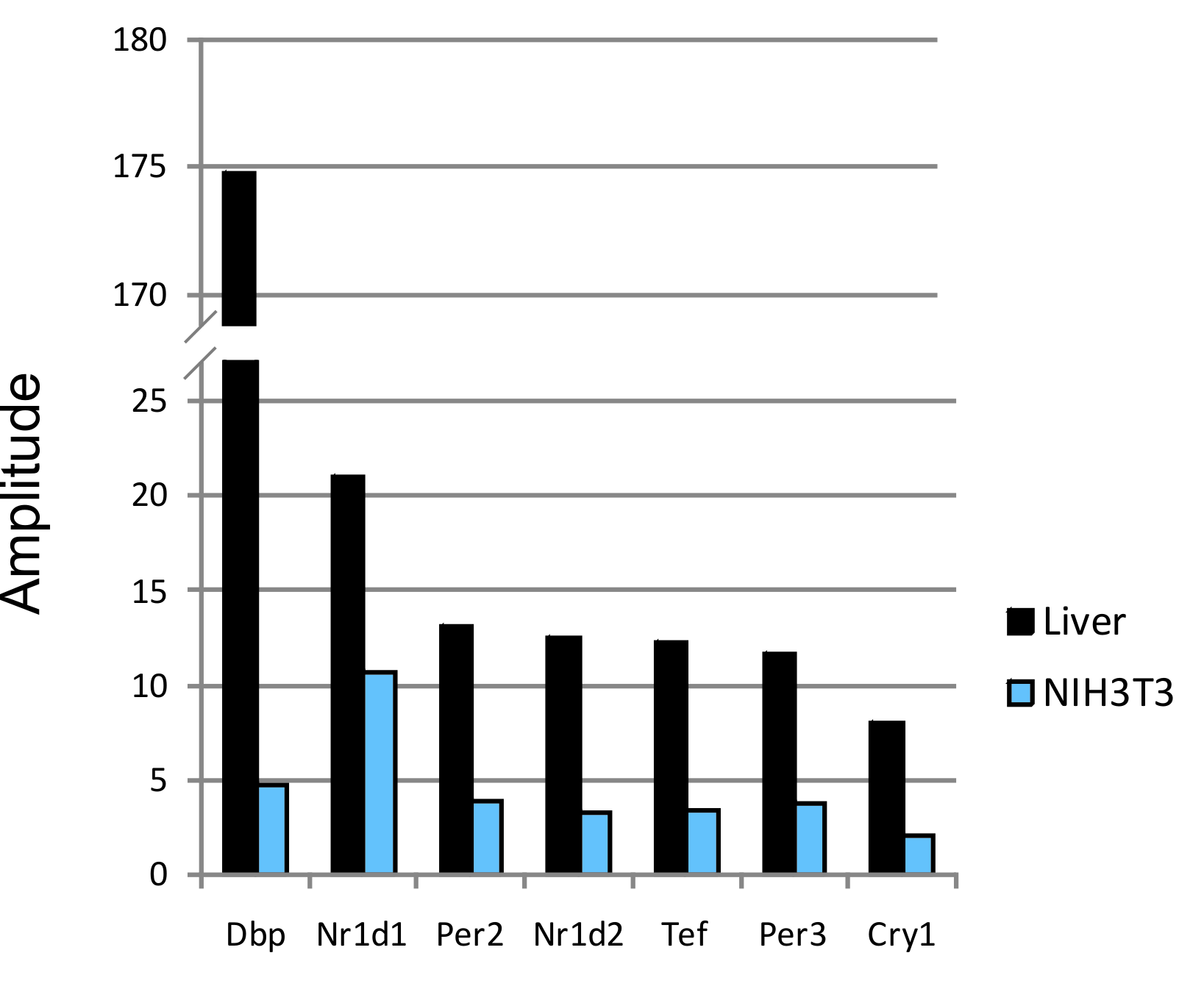

Supplement: Figure S9 — Amplitude comparison between liver and NIH3T3 cells. The amplitude of core clock genes was estimated by calculating the peak to trough ratio ( = percentile[0.95 , x]/percentile[0.05 , x]) and graphed alongside the amplitudes of the same genes in the liver. The differences in amplitude we observed were independent of microarray intensity between experiments as indicated by a comparison of the coefficient of variance (standard deviation/mean) for each probe (data not shown). (0.21 MB TIF) [file pgen.1000442.s009.tif]

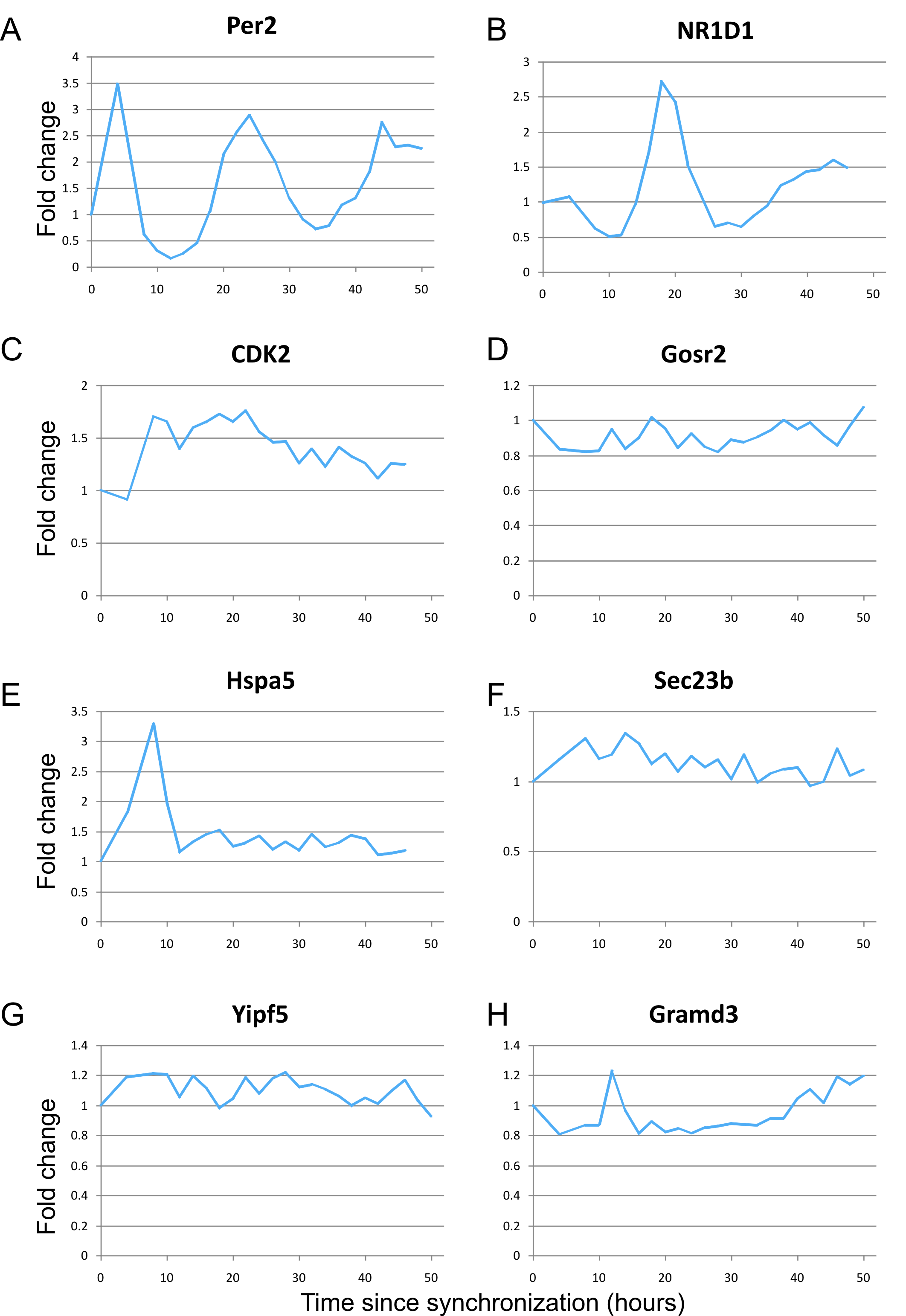

Supplement: Figure S10 — 12 h genes do not cycle in NIH3T3 cells. To validate the microarray profiling of NIH3T3 cells, a second time course of cycling 3T3 cells was collected every 2 h for 48 h. Quantitative PCR was used to measure the fold change of endogenous RNA which was plotted against CT time. Core circadian clock genes including Per 2 (A) and NR1D1 (B) oscillate with periods of approximately 24 h. In contrast, genes with 12-hour periods in the liver are arrhythmic (C–H). (0.68 MB TIF) [file pgen.1000442.s010.tif]

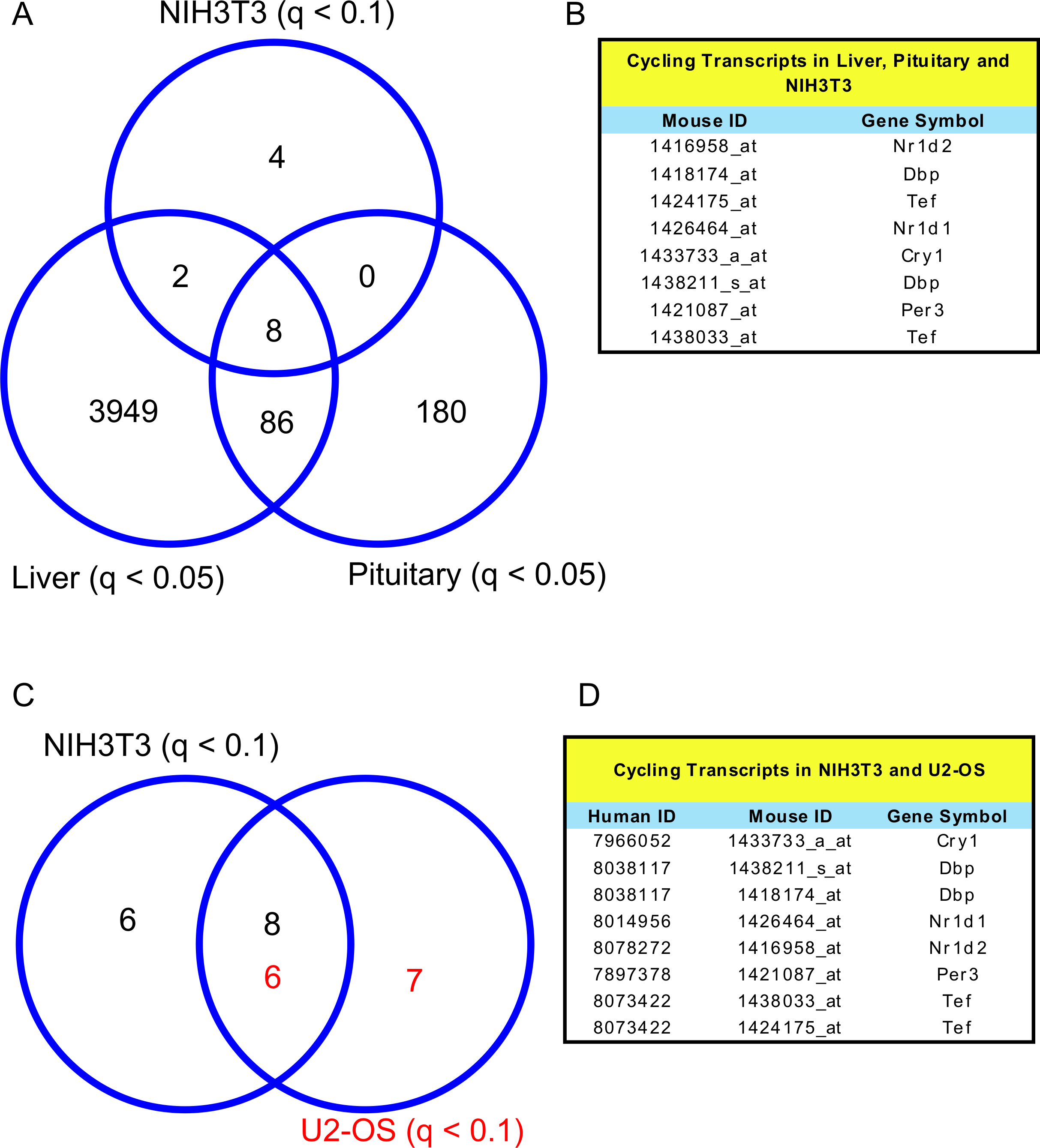

Supplement: Figure S11 — Comparison of Liver, Pituitary NIH3T3 and U2OS datasets. High temporal resolution profiling has been performed on samples from the liver, pituitary [24], U2OS and NIH3T3 cells. Cycling transcripts were detected in the liver and pituitary at a false-discovery rate of <0.05; rhythmic transcripts in U2OS and NIH3T3 cells were identified at a false-discovery rate of <0.1. The number of cycling genes common to each group was plotted as a Venn diagram. In (B), the number off cycling genes common to NIH3T3 cells (in black) and U2OS cells (in red) were plotted as a Venn diagram. (0.75 MB TIF) [file pgen.1000442.s011.tif]
